# Reflections on different governance styles in regulating science: a contribution to ‘Responsible Research and Innovation’

**DOI:** 10.1186/s40504-015-0026-y

**Published:** 2015-08-11

**Authors:** Laurens Landeweerd, David Townend, Jessica Mesman, Ine Van Hoyweghen

**Affiliations:** Radboud Univeriteit Nijmegen FNWI-ISIS & TUDelft, TNW-BT-BTS, PO box 9010, 6500 GL Nijmegen, the Netherlands; Department of Health, Ethics and Society, Maastricht University, PO box 9010, 6500 GL Maastricht, the Netherlands; Department of Technology and Society Studies, Maastricht University, PO box 9010, 6500 GL Maastricht, the Netherlands; KU Leuven, Life Sciences and Society Lab, Centre for Sociological Research (CeSO), PO box 9010, 6500 GL Leuven, Belgium

**Keywords:** Policy, Governance of S&T, Technocracy, Public participation, Ethics expertise, ELSA/ELSI, RRI, New and emerging science & technology

## Abstract

In European science and technology policy, various styles have been developed and institutionalised to govern the ethical challenges of science and technology innovations. In this paper, we give an account of the most dominant styles of the past 30 years, particularly in Europe, seeking to show their specific merits and problems. We focus on three styles of governance: a technocratic style, an applied ethics style, and a public participation style. We discuss their merits and deficits, and use this analysis to assess the potential of the recently established governance approach of ‘Responsible Research and Innovation’ (RRI). Based on this analysis, we reflect on the current shaping of RRI in terms of ‘doing governance’.

## Introduction

Under the influence of scientific positivism, the latter part of the 19^th^ century and the first part of the twentieth century were dominated by the societal belief that ‘science and technology development’ was rather synonymous with ‘social progress’. Its governance was left to professional scientists’ self-regulation, especially in the field of medicine (Krause 1996). The 1947 Nuremberg Code, drafted as a response to the medical experiments performed under national-socialism, can be seen as a first international policy attempt to define moral criteria for scientific conduct (Annas & Grodin 1992). The code was drafted specifically in relation to medical research on human participants, and other areas of research were not kept in mind in its drafting. It was further developed in the Helsinki Declaration (2012) and its subsequent revisions, again in relation to medical research with human participants. In the ensuing decades, the self-regulation approach in many other areas of scientific and engineering began to be challenged (OECD 1980, Braun et al. 2010). In the late 1970s and 1980s, this led to shifts in governance from self-regulation to external regulation.

Governance we understand to be the set of processes by which it is taken that stewardship over, in our case, science and technology practices (research, innovation, etc.) ought to be organised in continuous calibration with those practices. It involves policy makers, researchers, industry and civil society groups and nongovernmental actors in society. It includes, but does not exclusively refer to formal governing practices. Further, governance does not always calibrate to the full range of interests in society, but often only to the interests of particular groups, by diverting responsibilities whilst blurring interest-based motivations.

The first shift away from purely code-of-conduct-related regulation in governance created a ‘technocratic’ style of governance: structures created mostly through law that regulate science and technology through independent peer evaluation of science, focusing primarily on risk and risk assessment. A second shift away from self-regulation introduced an institutionalised place for ethical review alongside scientific adherence to such review. This broadening of the review of science and technology occurred, for example, in the European Commission Framework Programme research funding, where early EU governance strategies (mid 1990s to early 2000s) included experts on Ethical, Legal and Social Issues (ELSI) in addition to scientific expert based information in reviewing proposals competing for funding. Due to the perceived democratic deficit of the European Union, these two shifts were paralleled by a third shift in governance of science and technology While the latter shift already started in some European countries in the early 1980s, it came to be institutionalised formally in Europe in the 2000s. Dealing with approaches to facilitate the voicing of public opinion and representatives of NGOs, such as consensus conferences, public hearings, citizen forums etc., they were further developed and given an institutional place (see, e.g., the 2001 White Paper of the European Commission (European Commission 2001)).

New lines of research and innovation have emerged in the past decade such as nanotechnology, synthetic biology or human genomics. They promise to resolve global, societal challenges such as climate change, energy security, the economic crisis, and the accompanying global health challenges through the development of sustainable biofuels and biomaterials, home defence innovations and cheaper medication. Some of these developments are considered as positive (e.g. medical advances, greater mobility for members of society, cheaper and more sustainable sources of energy-production and usage), while other developments give rise to societal concern and public distrust.

To better anticipate societal needs and concerns, and to guide the societal embedding of such new scientific developments, a new governance approach, “responsible research and innovation” (RRI) has been proposed in the past years within the context of European Commission projects for research and innovation (Hoven Van Den et al. 2013; Zwart et al. 2014). It bases itself on a variety of approaches that can be captured under the heading of ‘responsible innovation’, and questions related to societal concerns over research and innovation can be treated within the frameworks offered within its reach (Stilgoe et al. 2013). It creates a platform where social deliberation can take place about relevant issues, and where concerns, needs and desires can be discussed and negotiated. Responsible innovation is the generic term for a conglomerate of new approaches in the governance of science. For example, the term ‘Responsible Research and Innovation’ (and ‘RRI’) is a major conceptualisation of a new governance in the context of the current European Commission research funding programme and its innovation platform *Horizon2020*, incorporating four dimensions: anticipation, reflexivity, inclusion and responsiveness (Stilgoe et al. 2013).

RRI is a specific European strategy to implement such a structure. In 2012, DG Research and Innovation of the European Commission published a three-page guide to explain RRI (European Commission, DG Research and Innovation 2012). It has six key principles: ‘engagement of all societal actors’; ‘gender equality’; ‘increase scientific knowledge and understanding in the population’; ‘free online access to results of publicly-funded research (publications and data)’; adherence to ethics ‘to adequately respond to societal challenges’ and ‘as a way of ensuring high quality results’; and, finally, ‘governance’, described in the 2012 document as an overarching element encompassing the other elements, stating:

“Policymakers also have a responsibility to prevent harmful or unethical developments in research and innovation. Through this key we will develop harmonious styles for Responsible Research and Innovation that integrate public engagement, gender equality, science education, open access and ethics” (European Commission, DG Research and Innovation 2012, pp 4).

Reading explanations such as that of the Commission, it is clear that RRI is considered a new approach to the governance of science and technology that requires some clarification.

### Aim and methodology of this paper and work

From the approaches to science governance that are discussed in the Report of the Expert Group on Science and Governance to the Science, Economy and Society Directorate (‘Taking European Knowledge Society Seriously’, 2005), three major groups can be identified as prominent over the recent past in the ‘who?’ of advice in governance: first, those with scientific, technological expertise, with their input being guided by legal expertise; second, those with ‘ethico-legal’ expertise ; and third, those included as part of ‘public participation’. In this paper, we review the three styles of governance that can be associated with these ‘who?’s. We argue how they co-constitute the current governance of science and technology in Europe: earlier styles have not been replaced; each new style of governance has added an extra layer in governance that takes on the dominant expression to (seek to) ensure public trust and confidence. By reflecting on the merits and deficiencies of the different styles of governance of science and technology in Europe, we further aim to inform the shaping of current and future RRI-initiatives. The research for this paper has been undertaken as part of the EC FP7-funded project Ethics in Public Policy Making: The Case of Human Enhancement (EPOCH).

Our analysis is based on a review of literature and interviews with experts with different relevant professional backgrounds involved in the European governance of science and technology. We set out with a data collection that focused on how the governance of science and technology came to be organised in Europe. We analysed a variety of resources on governance approaches of science and technology, which we identified in policy documents and publications from a wide variety of journals, conference books, project reports, websites and monographs. We included documents from a wide range of disciplines and different experiences, including:

1. political theory and expert literature on governance of science and technology and its historical background;
2. legal documents and literature on governance of science and technology;
3. sociology, and science & technology studies;
4. bioethics literature on governance of science and technology (including literature both from academic bioethics as well as their various contributions to policy documents);
5. secondary literature on the role of bioethics in governance of science and technology (including literature in which bioethicists analyse their own field of work and papers from the area of governance theory);
6. literature on public participation (including expertise such as sociology, bioethics, science and technology studies, and political theory); and,
7. literature on socio-cultural representations and imaginaries on science and technology and their potential for governance (including literature from philosophy, and science and technology studies).

Our data collection was based on keyword searches through general search engines like Google and keyword searches through Maastricht University’s e-library, which also contains the option of a meta-search through which more than one database can be searched. Databases included JSTOR, Medline, Oxford Journals, Swetwise, Kluwer, etc. We used keywords such as ‘science governance/science policy’, ‘ELSI/ELSA’, ‘RRI’, ‘responsible governance’, etc.

We strengthened our preliminary literature findings by conducting a series of open qualitative targeted interviews with experts in science governance with a variety of backgrounds (ethics advisory groups, research ethics experts, experts in science policy, etc.). We used the interviews to gain critical input on our preliminary literature selection on governance approaches to science and technology, and conclusions on our analysis of that literature. Through the interviews we also gathered further input on the nature of governance as well as on the specific role of ethics in the governance of science and technology. Thus, as well as forming a ‘completeness check’ on our literature overview, these interviews also provided a more informal input on issues in past and contemporary science governance. Our approach to the interviews was based on qualitative expert interview techniques, with a preference for semi-structured interviews. The interviews were recorded on a voice-recorder or, when the interviews were conducted through Skype, with the aid of a freeware programme that records Skype conversations (with the consent of the interviewees).

The interview questions were subdivided into two sets. The first set of interviews explored views on the current state-of-the-art in studies of governance of science and technology. The second set of interviews was based on the results of our literature review and explored the perspectives of the Interviewees on the strategies and styles of governance found in the literature. Considering the wide range of issues involved, we decided to interview individuals with a diverse set of professional perspectives on these matters. Our Interviewees had backgrounds in one (or more) of the following disciplines: philosophy, policy-making, science and technology studies, ethics of new and emerging science and technology, future scenario building, science communication, and sociology. From July 2011 to February 2012, the first author (Landeweerd) held interviews with 10 international experts in the field of governance of science and technology. These were based on – and in some cases held at - a number of international conferences on governance of science and technology that he attended. Four of these ten are directly quoted in this paper. The others contributed mainly to this paper through their advice on literature.

### Three styles of governance of science and technology

Governance can be considered as “the complex of formal and informal institutions, mechanisms, relationships, and processes between and among States, markets, citizens and organizations, both inter- and non-governmental, through which collective interests on the global plane are articulated, rights and obligations are established, and differences are mediated” (United Nations Economic and Social Council 2006). But this is not achieved easily: it is difficult to determine which interests and actors should be included in the process or activity of governance. Below we will present a critical reflection upon the different styles of governance. In particular, we seek to identify issues that have been overlooked in these strategies and to give an account of how governance for the relevant areas in science and technology is currently shaped and in which ways it could be developed.

### A ‘technocratic’ style of governance

In the ‘technocratic’ style of governance a specific format for decision-making is dominant. This style holds two aspects of technical regulation: scientists and technologists as assessors of acceptable risk; and law and lawyers as framers of governance procedures, for example in giving suggestions for changes to legal frameworks, suggestions for self-regulation or suggestions for new regulations. The sociologist Callon refers to the process that underlies this style as a ‘double delegation process’: the power to decide is delegated here from citizens to parliament (e.g. through legislation) and from parliament to scientific communities when specialised expertise is deemed necessary (Callon et al. 2009). A focus on risk and risk assessment, rather than on moral or other questions when asking whether the science or technology ‘ought’ to be pursued is the core of this approach. Law, as a product of citizen’s democratically elected representatives, sets further boundaries within which the science and technology community must operate to evidence its self-regulation, and thereby to ensure trustworthiness. In this technocratic style of governance, scientific expertise is framed as neutral, rational and well-informed, and public opinion as irrational, pitting the two against each other (Wynne, 2006). This style of governance comes from a view of science as rational: neutral science is seen to provide valid knowledge, and therefore provides input for governance that is necessarily superior to any non-scientific (i.e. irrational, biased, etc.) forms. The idea that the non-expert, the public in particular, may be biased due to a deficit of knowledge and a lack of expertise on the subject is typical to this style of governance. It is generally known as the ‘deficit approach’ (Stirling 2008).

This style is often associated with ‘governing´ (top down, centralised, of science and technology on the basis of invited advice from scientific expertise), as opposed to ´governance´ (bottom-up, decentralised) (Callon et al. 2009)). In this style, the law is usually the central procedural vehicle to deal with issues, whilst the substantive focus is predominantly on risk and risk assessment. The law is regarded as an effective instrument and provider of neutral information (with a similarly positivist understanding of the function of Law in society). Law operates to give structures within which science and technology are not only constrained against risk, but within which science and technology’s own internal competitions are shaped by patents and intellectual property rights (Howells et al., 2007). Thus, at the international level, the human rights agenda (Universal Declaration of Human Rights 1948) enshrines human dignity and autonomy as bedrocks for the avoidance of risk or the individual’s right to be made aware of risk and to accept or decline risk, and stewardship, as either a basis for making risk assessments on behalf of others, or in the development of international agreements about environmental risk assessment (United Nations 1992). Due to the initial dominance and societal resonance of the technocratic style of governance, risk assessment was long considered central for governance of ethical issues in science and technology (Felt & Wynne, 2007).

The technocratic governance style remains visible in Law. Basic rights paradigms are constructed through international, European and national agreements and regulations. Thus, medical research, for example, is regulated by the Helsinki Declaration at the international level (Helsinki Declaration 2012), which is translated (at a technical level) into the work of individual doctors and medical researchers primarily through contract, or for example, through the Clinical Trials Regulation of the EU. In biomedical science, for example, it is also regulated generally in relation to biomedical research through the Oviedo Convention, and in national laws regulating research practice. The network of law extends into international agreements about the processing of personal data, which finds European expression in the Data Protection Directive. This is translated into national law across Europe, and by its legal nature, tends to remain technocratic in its approach.

In the technocratic approach, science is regarded as a linear, rational and goal oriented process. Societal impacts are typically considered and addressed during the dissemination or implementation phase and are focussed on particular technologies. In this perspective, ethical and public accountability is also promoted through ‘scientific technocratic’ approaches, whilst societal opinion is seen as potentially biased.

#### Deficiencies in the technocratic style of governance

A shortcoming of the technocratic style is that political decisions are reduced to purely technical decisions, for example on the basis of a calculated risk estimate of the usage of chemical substances. In practice this does not work since such decisions always also imply normative reasoning. Furthermore, due to the complex nature of contemporary, interdisciplinary science and technology, which always involve issues of uncertainty, its authority within society has been declining. (Callon et al. 2009)

Dominance of the technocratic style of governance led policymakers to hold too narrow a focus on risk assessment and incentive management. Genetic modification of foods, for example, was generally not accepted by the public, but for reasons other than risks to health such as unnaturalness (Bauer 2002). There are other legal and moral questions that influence public opinion, and merely harbouring societal acquiescence is not sufficient legitimacy. These include problems related to patent law around technology and knowledge transfer to developing countries and emergent economies. Next to ‘hard’ issues in governance of science and technology such as risk assessment, soft issues such as naturalness, playing God, techno-scepticism and environmentalism also play an important role in the public’s acceptance of science and technology (Swierstra & te Molder 2012). Often these are ignored in the technocratic style due to their perceived ‘irrationality’ (Carreda 2006).

Currently, many scholars perceive another problem in the technocratic style of governance (Nicolosi & Ruivenkamp 2012; Sutcliffe 2011; Flipse et al. 2014). Societal debate gets to be triggered by products at the end of the development chain, rather than during the processes of scientific progress and technology innovation themselves, when things can still be shaped and steered in different directions. Thus, it has become commonly accepted amongst experts of governance and policy of science that research and innovation trajectories would be of a higher societal quality if researchers and funding agencies consider the wider societal implications of the innovations that are triggered by their work from the very outset Nicolosi & Ruivenkamp 2012; Sutcliffe 2011; Flipse et al. 2014). After all, without sufficient anticipation on the values, needs and concerns in society, a research and innovation trajectory is bound to meet with public resistance. This means that such implications need to be considered not only in the context of “applications”, but before, *during* the process of agenda-setting. The problem, however, is how to operationalise this idea and trigger such a complex process.

A related critique was already voiced quite some years ago in regard to legal expertise (Forester & Morrison 1991). Due to the increasing complexity of both innovation processes and their societal impact, the law is acknowledged to be limited in its capacity to deal with issues of public trust. As Forester & Morrison state:

“technological change penetrates society faster than we can form new attitudes, reach new consensuses, or adapt our legal and ethical codes” (1991, pp 299).

The pace of new and emerging science and technology (NEST) is problematic since legal frameworks and ethical discourse simply cannot adjust quickly enough to the new dilemmas brought along with research and innovation. Forester & Morrison consider the fact that science and ethics are still very much regarded as separated realms as a cause to this issue. In their view, a further integration of democracy, ethics, engineering, science and policy making could resolve this issue.

### An ‘applied ethics’ style of Governance

To facilitate ethical and social acceptability in a more strategic fashion and to generate legitimacy for research and innovation, new approaches to the governance of S&T have been defined. The civil rights movement, specifically, formed a push to the implementation of this style. The ensuing approaches were based on input from ethical experts from the fields of applied ethics and bioethics as well as socially engaged scientists with a specific experience and interest in ethical issues related to science and technology. Ethics in governance of science and technology arguably holds an influence in an advisory capacity on moral issues that are intrinsically connected to science and technology but also as mediator with regard to the shape of platforms of debate, in terms of transparency, democracy and trust.

James Watson, co-discoverer of the double helix structure of the human genome and also a leading scientist in the human genome project, expected the science-society contract to be revisited (Zwart & Nelis 2009). He was concerned that the mapping of the human genome challenged traditional values, and thus saw research to its ethical, legal and social implications (ELSI) as desirable. In the early 1990s, the European Commission adapted a similar research programme: ELSA (ethical, legal and social aspects) (European Commission 2008). It is a programme that, apart from scientific and legal experts in the technocratic style discussed earlier, also involves professional ethicists. It is specifically in this context that ethics came to be institutionalised as a normative instrument to justify law and regulation (Tallacchini 2009). ELSA-research initially aimed at giving advice as an observer of scientific research and technology innovation, from a discursive distance to actual lab research or industrial practices, but this changed over the years, and finding means to integrate the efforts involved with such practices became increasingly important.

In its institutionalised role, ethics expertise is regarded as a “‘neutral’ normative tool, endowed with the potential to speak for rationality” (Tallacchini 2009, pp 281). In this normative capacity, the field is regarded as “the determination, so far as that is possible, of what is right and wrong, good and bad, about the scientific developments and technological deployments of [at first predominantly, but later on not exclusively] biomedicine” (Callahan 1999, pp 276).

An example of the prominence of ethics as a governance tool can be found in the evaluation processes for Science in Society” programmes research applications. Over consecutive ‘Framework Programmes’, ethical review has become more prominent; proposals for research, once they have been evaluated positively for their scientific quality, are assessed for their ethical quality by a panel of ethics experts drawn from across the EU (Tallacchini 2009). The stated role of ethics expertise in governance is to provide input on the moral delimitations of science and technology. In practice, ethics expert advice is often used in governance as a way to *de-politicize* science and technology. For many expert ethics committees, this often means that the main agenda is to come to consensus, to find solutions to issues; that is, ‘closing up’ controversies on normative issues (Stirling 2008).

#### Deficiencies in the applied ethics style of governance

Ethics has become an important normative ‘instrument’ for European policy making (Tallacchini 2009). This has led to criticism of such approaches as being the handmaiden of science and technology rather than critical observers and assessors of their societal impact (Turner 2012, as well as Interviewee 1). The institutionalised role of bioethics as a source of advice on ethical issues in the governance of science and technology is not regarded as unproblematic, as we will summarize below.

A main problem related to applied ethics is the place of moral principles and moral practices. As the main author of this paper has argued elsewhere, the problem lies in the relation between abstract principles on the one hand, and moral agency in practice on the other (Landeweerd 2009). A variety of approaches has been proposed to resolve this issue, including applied principlism, casuisty, specified principlism, and pragmatism. As a result, whilst having become embedded in institutional practices, applied ethics has gradually become increasingly focused on the delimitations of the moral debate rather than its content (Habermas 2003).

It may be questioned why a specific ‘ethics expertise’ is needed to assess the moral acceptability of specific new technologies, and why an expert view is superior to a lay opinion. Interviewee 2, holding a background STS/governance theory, expressed this view clearly:

“So in that sense I am sure bioethicists have now become the professionals in the field. They are analysts that analyze a problem from a particular point of view, but I am unsure what this tells me about the expertise they have. I mean, the expertise they have in making some futures possible or impossible […]. Like by vocation they decide where […] we can go with research, but […] what is the kind of special knowledge they hold, by which I do not claim that they cannot do an analysis from a philosophical point of view. But this is not the same thing than having an expertise on […] making choices where society should go or should not go.”

In a similar vein, the potential of ethics-expertise in giving prescriptive recommendations was criticized by some interviewees. The issues that are at stake in governance of science and technology are often normative assessments, calling forth for plural conditional advice instead of normative prescriptions. As Interviewee 1, also holding a background STS/governance theory, said:

“So […] if every expert committee, whatever the discipline, was charged with the responsibility not just to give a prescriptive recommendation, like “we think X, and therefore we recommend that Y”, but actually would say, ‘under condition A, it follows that X is the conclusion and therefore we [recommend that] Y; under conditions B actually Y is the implication’ [and therefore we recommend Z]. [One should] say: under these assumptions [one should] do this, under these assumptions [one should] do that. The actual assumptions are not matters on which we are experts. They are political judgments; they are ethical considerations, value judgments. […] There is no such thing as expertise on what is politically justif[iable]”.

For Interviewee 1, the institutionalised role of ethics for policy making on science and technology should be regarded critically:

“[…] for policy makers [expert ethics advice] is a way to implement policy. So apart from scientific justifiability, expert advice is often used to ‘get things through’”

Eckenwiler and Cohn (2007) trace some of the most important concerns in bioethics with regard to this institutionalised role. They assess how ethics, as ‘voice of morality’, copes with the tension between bioethics as an actor on behalf of morality and bioethics as an institutionalised party that has become part of the very field it purports to assess. Specifically with regard to the latter, the role of bioethics may be problematic: in a sense that is very similar to the decline of the authority of expertise in what we termed the ‘technocratic style’; bioethics expertise may be as much the servant of different actors in science and technology as its ‘neutral’ arbiter. And even in that latter capacity, it can be questioned whether the opinions held, and the arguments provided, by a bioethicist are any more justified than any other expert or lay person.

Ethics expertise is not only considered as a useful device to inform policy agendas on issues of legitimacy and acceptability, these agendas are also often pre-formatted, giving way to the problem of framing. Ethics advice committees, for example, are often set up within a pre-defined set of aims: coming to consensus, finding solutions to issues; that is, ‘closing up’ controversies on normative issues. As Interviewee 2 reflected on his experience in an advisory commission:

“[…] the decision maker is surrounded by their gatekeepers. The power of the gate keeper is determined by conditioning what goes in. […] So you know, even within that very small location there was a micro dynamic playing out. […] the message we get, as academics, is that ‘don’t just tell us what to do, […] tell us to do the thing we want to do’.”

In this sense, ethics-expert advice is used as a way to *de-politicize* science and technology. This means – above all – that agendas of ethical expert committees are pre-formatted to the very aim of getting consensus. Getting closure and enforcing consensus may in some cases be a legitimate target, but it is questionable whether this should be the central goal for institutionalised ethics. Interviewee 2 stated the following about the dynamics of ethics advisory committees and the issue of indirect coercion to adjust recommendations to predefined agendas:

“What in fact happens is everyone knows that are you serious. I mean, if you are all working out there for two hours to get a consensus, and you [are] seriously [expressing your views], you will be, yeah, certainly not getting an award at the end of your career. You won’t be, maybe, put out of the committee […], but there are also other very powerful disincentives.”

As Interviewee 2 continues:

“[…] They [referring to policy makers] wouldn’t pick up any of your recommendations. They have to fit in particular pre-made set of possibilities. So, and to negotiate these pre-made sets [referring to the pre-set restrictions for advisory committees] would be the really important issue here.”

The latter critique on the pre-formatted framing of ethics advice in governance of science and technology is also reflected in the critical assessment of different strategies to implement ethics in European research programmes, such as the Science in Society programmes and the envisaged increased embedding of ethics in research, as envisioned for *Horizon2020*. As stated by Interviewee 3 on a question related to ethics:

“In research projects with an embedded ethics Work Package you have a very close relation to ongoing projects which is good, on the one hand. But this poses some problems in that you are always very close to your partners and in that sense institutionally bound to them”.

In this sense, the independence of ethics input may be put at stake when ethics is too embedded in science research projects. As Interviewee 3, who held a background in philosophy and bioethics, stated in elaborating on the place of ethics in policy:

“[…] if you would think about an ethical analysis, you might want to have that more remote from the current project and practices of science and technology”.

The issues of proximity and the problem of ‘external influences’ are already present in the staging of new research projects on ethics.

A further problem is whether ethics advice, when it has been drafted, will actually be used. The European Group on Ethics in Science and New Technologies (EGE) plays an important role in the formation of opinions and strategies for the European Commission. But, as Interviewee 4, with a background in policy of science and technology, stated with regard to the EGE:

“[…] the EGE provides a lot of interesting overviews, but it seems to me a little bit unclear what the governance and what the political status of the EGE statements are. I mean they go first of all to Barosso and the commissioner and then they are published so in that sense they are part of the EU administration and also again there the question is what kind of advice is that in relation to what governance then is enacted […]”

In the case of the EGE, the identification and selection of the EGE members is made on the basis of an open call for expressions of interest, and is guided by support of a member of the European Parliament, providing for its democratic legitimacy. Whether its advice is taken up however, remains unclear. The problem is that governance necessitates a broader approach than merely a process of double delegation (Callon et al. 2009).

A final critique on the role of ethics in governance of science and technology is more content-related. In a report for the European Commission, René von Schomberg states that contemporary ethical theories cannot capture the ethical and social challenges of scientific and technological development, due to its focus of intentional individual agency rather than unintentional, collective, societal effects (Von Schomberg and European Commission, DG research 2007). Drawing on the paradigmatic examples of Eichmann and Oppenheimer, von Schomberg demonstrates the rationale behind individualist approaches to ethics but he also argues that it is impossible to pinpoint who is responsible for issues such as nuclear weapons proliferation, ozone depletion, or global climate change. Whilst some scientists may feel responsible, they are often not competent to act, necessitating, what he calls, a collective ethics of “co-responsibility”.

### A ‘public participation’ style of governance

A loss of trust in science, technology, politics and ‘top-down’ governing has also spurred ‘bottom-up’ activism, or spontaneously emerging public engagement from citizens themselves demanding their voice in governance of science and technology (Bucchi & Neresini 2007). From the late 1970s, more proactive approaches to governance were developed to directly involve citizens in decision making on science and technology, be it for surveying public opinion, for consultation, or for direct democratic decision making. Examples include the response to the use of Agent Orange in the Vietnam war, public resistance against the development and storage of nuclear weapons, and activism against abortion. Legitimacy of science policy is not merely guided here by the rationale behind science: it also needs to be guided by values of the decision making processes as such, including the principle of transparency and the need for democratic policy structures. Apart from the moral status of the decisions made, the demand for transparent and democratically informed and supported policy that is pushed by such instrumental values has necessitated inclusion of citizen’s perspectives (Wynne 2006).

Over the course of the past 30 years, different mechanisms have been put to practice in different regions and countries to reach a higher level of public participation. These include citizen juries, citizen panels, consensus conferences, planning cells, deliberative polling, focus groups, consensus building exercises, surveys, public hearings, open houses, citizen advisory committees, community planning, and referenda (Funtowitz & Ravetz 1993). This multitude of approaches, strategies and formats is applied in different settings, with different justifications and purposes. They all aim to consult and involve the public, either normatively argued, as a recognition of basic human rights regarding democracy and procedural justice, or from a instrumentally argued recognition that implementing unpopular policies without involving the public may result in widespread protest and reduced trust in governing bodies. Next to the gathering of normative views amongst the public, public participation is argued substantively to be useful as well: knowing in advance what society wants is supposed to enhance the quality of innovation processes, and thereby lessen the chance of public adversity (Stirling, 2008). In other words, being aware of public opinion and public preferences would aid in enhancing the success rate of innovation processes and of the landing of the resulting products in society. This rather presumes that ahead of innovation, the public can foresee novel developments and inventive steps – which, by definition, are outside their current experience. Did we know that we wanted the iPhone before the iPhone was developed?

Over the past decades, the debate over the role of the public in creating policies on issues of science and technology has increased (Rowe & Frewer 2000). Public input is now incorporated in national and international governance and risk management in both formal and informal ways (Klauenberg & Vermeulen 1994). This move can be seen as a response to how public opinion drives political and governmental choices. This also follows for the European Commission. In 2001, the White Paper of the Commission of the European Communities on “European Governance” was published after a comprehensive consultation process to gain back public confidence after the scandals of the Santer-Commission and in order to mitigate the democratic deficit of European policy-making (Commission of the European Communities 2001). The Paper acknowledged the importance of public participation for European governance of science and technology. It particularly addressed the growing criticism of a lack of democratic legitimacy of the EU during the 1990s, pointing out the “mismatch between the concrete achievements of European integration on the one hand and the disappointment and alienation of ‘Europeans’ on the other” (Armstrong Kenneth 2001, pp 119). The Paper aimed to marry theoretical accounts of participatory forms of governance with a practice-oriented account of public or civic participation in governance of science and technology.

The development of novel technologies and their introduction to society could thus be threatened by public adversity, an issue to which the technocratic style of governance could not respond. It was increasingly acknowledged how new forms of science and technology were unpredictable in nature, and how relevant policymaking could not merely be an issue of risk calculus. The inclusion of public values and a pluralist notion of ethical principles through participation now complements forms of ethical technology assessment. But their practical operationalisation is being regarded with increasing criticism as well (for an early example of such criticism see Cooke & Kothari 2001).

#### Deficiencies in the public participation style of governance

Public participation, although well established in governance of S&T, is criticised first of all for suffering from a lack of evidence over its quality and impact. Rowe and Frewer (2000) state that there is a general lack of empirical consideration of the quality of public participation methods. They claim this deficit emerges from confusion as to the appropriate benchmarks for evaluation. They argue that the most appropriate method of public involvement depends on the specifics of particular situations.

A second criticism of the public engagement style of governance concerns the democratic legitimacy of public participation initiatives (Jasanoff 2005; Leach & Scoones 2006 Lafont 2014). In the literature and our interviews, several drawbacks in this regard were reported. First, the question was raised to what extent the people who participate in these initiatives are representative of the public. As Interviewee 4 for example argued:

“[…] the question is what kind of democratic legitimacy this discourse has. […] you get only a perfect cut of the population: the people who are willing to participate in those discourses might not be an average of the population but only a group of people with specific agendas.”

Further, the extent to which citizens are invited and willing to participate in decision-making processes for science and technology varies by country and its established political culture. Jasanoff sketches out concrete and existing forms of public participation throughout different institutions and societies. Using the examples of the U.S., Britain, and Germany, she shows how different political traditions in different countries employ different forms of public reasoning on science and technology and hold different attitudes to the ethical, legal and social issues connected to biotechnology and how they hold different views on public participation (Jasanoff 2005). These ‘civic epistemologies’ show the characteristics of the specific institutions they are part of and how the latter are decisive of how an issue is framed as a policy issue. The concept of ‘civic epistemology’ serves as a useful tool to compare different ways in which governance of science and technology is embedded in specific local institutions and their cultures and traditions.

Apart from self-selection processes of public participation, another criticism about the democratic legitimacy aspect is whom the ‘public’ engaged in the process was supposed to represent, or, indeed, did represent. According to one of our interviewees, the staging of specific public representative groups, such as NGOs, raises the question of whether they represent ‘the public’. As he argued:

“Unfortunately there has been a tendency to take any NGO or other outspoken interest group as representative of the public, overlooking that the public is multi-faceted and complex. This results in bias from providing a too strong influence for these groups, neglecting the public as such” (Interviewee 3)

These latter accounts about the extent to which public participation devices can represent the public, and then, which kind of publics, raises the important issue of who the public is, and to what extent ‘the public in general’ can be represented at all.

Finally, another more fundamental criticism that has been reported concerns the problem of framing in public involvement initiatives. Authors such as Felt and Jasanoff, although acknowledging the need for public participation, have indicated problems that may arise with these public involvement governance tools (Felt et al. 2009). According to them, public participatory exercises are always framed in specific formats and ways that are useful to specific actors. In other words, deliberation platforms or public participation initiatives are pre-formatted by broader or other political actors and agenda-setting. As Interviewee 1 reported:

“So I think, deliberation is a really important element in making political choices. But it cannot be a replacement for making political choices. […] It is not the locus of politics.”

In line with this, other interviewees questioned the status of these kinds of initiatives, referring to examples where outcomes of public engagement initiatives have been simply become archived by politic makers. As Interviewee 3 said:

“So the public discourse, if it is properly organised, is a good thing in itself, of for having active and knowledgeable citizens, so in that sense you couldn’t have too much of that. But you have to be aware and you have to clarify what the status of these kinds of efforts is within governance, and policymaking.”

In line with the critique on the ethics-expert style, the public engagement style often gets to be staged as a form of ‘scapegoat’ for policymakers, as an efficient tool of *de-politicizing* science and technology. Its organisers are often not sufficiently aware of this. Interviewee 3 nicely summarised this tendency:

“Anyway, there is always GM crops debates in the back of the mind of the funders, that's what they are afraid of to repeat. And the STS and ethics community grab the opportunity assuming that all want participation and democratisation of S&T, deliberative democracy and so on. But it's a rocky road and the public is elusive to all.”

The question here is what is done with these public participation outcomes. In Europe, actors involved in governance of science and technology fail to escape the influence of more technocratic tendencies in governance, albeit under the guise of democratic “bottom-up” engagement. This may also be the reason why citizens participating in such exercises resist being framed as representatives of ‘the public’.

### ‘Doing Governance’ through RRI

‘Responsible Research and Innovation’ is an approach to governance of research and innovation intended to replace the existing ELSI approach. René von Schomberg, scientific officer at the European Commission, played an important role in the preliminary definition and adoption of the RRI concept (Owen et al., 2012; Rodrígueza et al. 2013). According to Von Schomberg (2011a), specific ‘normative anchor’ points (such as sustainable development; competitive social market economy; full employment and social progress; protection and improvement of our environment; no social exclusion, meaning social justice added to quality of life), need to be a basis for EU governance of science and technology. They need to be taken as positive triggers for innovation rather than negative constraints. An implementation of these elements means an assessment of what would be the ‘right impacts’ as well as what would be the right processes to carry policies to such right impacts. The latter necessitates the definition of acceptability-criteria such as quality of life, sustainability, next to criteria of social needs. In this sense, ‘ethical issues of science and technology’ should be broadened up to include topics and issues addressing community values and collective behaviour.

Besides von Schomberg, Hilary Sutcliffe, director of the think tank on responsible innovation ‘MATTER’, played an important role. He prepared a report on RRI for DG Research and Innovation that outlines the principles for such an approach (2011, pp 3). At the beginning of the report the following common understandings of RRI are summed up to include:

1. “ the deliberate focus of research and the products of innovation to achieve a social or environmental benefit;
2. The consistent, ongoing involvement of society, from beginning to end of the innovation process, including the public & non-governmental groups, who are themselves mindful of the public good;
3. Assessing and effectively prioritising social, ethical and environmental impacts, risks and opportunities, both now and in the future, alongside the technical and commercial.
4. Where oversight mechanisms are better able to anticipate and manage problems and opportunities and which are also able to adapt and respond quickly to changing knowledge and circumstances.
5. Where openness and transparency are an integral component of the research and innovation process.”

RRI aims to strengthen ex ante consideration of societal needs and ethical aspects in research and innovation practices, amongst others, through research funding programs related to, for example, public and stakeholder dialogue. To do so the RRI approach includes the formulation of criteria for the early appraisal of research and innovation (Stilgoe et al. 2013; Hoven Van Den et al. 2013, pp 12). It is expected to meet today’s challenges through international, innovative and trans-disciplinary research and the empowerment of research consortia, governments, industry and civil society. In this regard, researchers and funding agencies are expected to consider the wider societal implications of the innovations that are triggered by their work *from the outset*, rather than in the stage when completed products are introduced to the market. To be able to achieve these aims, RRI focuses on “embedded research” – by normatively involved social scientists in close proximity to the sciences and related industrial practices. To do RRI-research one needs to become part of the very processes one studies (Zwart et al. 2014).

Currently RRI is being embedded in the research and innovation strategies of the European Commission and has become an integral part of societal embedding of the commission’s Horizon 2020 programme. The uptake by the commission of RRI as a basic strategy for dealing with issues of good governance of research and innovation has led to enthusiasm in existing social scientific expertise on science governance to support the initiative (e.g. Guston 2006; Sutcliffe 2011; Von Schomberg 2011a, von Schomberg 2011b; Lee 2012; Owen and Goldberg 2010; Randles et al. 2012; Stilgoe et al. 2013) observe a tendency in these accounts that they are aimed at nurturing responsible governance first.

Just as later stage ELSA research and parallel initiatives, rather than normative assessments by scientific or ethics experts, RRI aims for integrating societal aspects in these initiatives ex ante, from the outset. RRI thus also embraces a stronger integration of ethics and societal aspects in research and innovation. However, such integration ex ante may lead, again, to problems of framing, defusing, and taming of critical debate and strategic legitimation rather than substantive legitimacy of policy. RRI may become a tool for technocratic purposes as much as any other approach in governance of science and technology.

There is also a notable difference between the stated goals of later stage ELSA research and RRI: in contrast with earlier approaches to governance of science and technology, RRI shifts its attention to innovation as a trigger for socio-economic progress (Rodrígueza et al.; van den Hoven et al.; Zwart et al. 2014) rather than a mere implementation of societal factors. This may steer governance into a direction in which private interests overrule public legitimacy, and uses integrative approaches for other goals than as goals in themselves. We thus see a need to state a caveat: RRI fits in with the idea of moving from ‘governing’ to ‘governance’; for those who applaud this move, governance, rather than locating the authority of decision at the level of policy makers, aims for an embedding of decision-making processes within practice itself. This however potentially damages the autonomy of the expert communities involved as well as the sovereignty of the public bodies (politicians, policy makers) that should guarantee public legitimacy of the choices made. In that case, cooperation between public and private, although it may seem to enhance societal embedding of R&D, may actually render public funding and the public interest sub-servile to private interests. Increasing the extent to which these interests serve public goals as well as private ones may be a positive thing, but this does not mean a voice for public interest is no longer needed. RRI can only be successful if it develops strategies to avoid an erosion of publicly delegated sovereignty.

There are several conditions that may contribute to this. First, approaches to governance need to move beyond the idea of governance as ‘quick fixes’ to ethical issues of science and technology. One needs to acknowledge that there are no clear-cut, well-defined and predictive/foreseeable solutions to be found. In this regard, Guston’s concept of real-time technology assessment (Guston 2002), as based on the work of Rip et al. (1995), might be a good process-based approach: Guston aims to direct social scientific findings on the complex linkages between society and science, to an enhancement of the value and capability of the sectors involved. In his opinion, such a connection has not been achieved sufficiently. His strategy is a joint programme between natural and social sciences that would lead to a “real-time technology assessment” combining fundamental understandings of the social, moral, political, and economic dynamics of knowledge-based innovation. Recently, the idea of real-time technology assessment is taken up and elaborated (e.g. Stemerding & Rerimassie 2013. Also Eric Fisher attempted to design an approach that meets the demands to go beyond the natural and social science divide as well as the ‘top-down’ and/or ‘bottom-up’ approach. He provides a methodology, “midstream modulation”, that facilitates the interaction between the natural sciences, the social sciences, and ethics, with the aim to yield a more socially robust approach to research and innovation (Fisher et al. 2006). As such, it contributes to the debate between empirically descriptive ethnographic approaches to science and technology practices in the social sciences, and approaches that call for a more ‘interventive’ and normative steering of science and technology, whilst taking into account the need for marrying two problematic forces in the debate: technocratic views that aim to inform society on the yields of science and technology, and designs for upstream engagement to facilitate societal influence on science and technology.

Secondly, acknowledging complexity means that governance should be less about defining clear-cut solutions and more about making explicit the political issues that are at stake in science and technology. In this sense, governance becomes a process in which the political nature of science and technology is made explicit, where concerned actors express that there is *de facto* not one, single answer. ‘Doing governance’ implies the space for making explicit what is moving all the different (kinds of) stakeholders on issues of science and technology. This means focusing less on ‘decision-making’ and more on identifying the shared values and interests we have in the issues on the table; a focus on collaboration and dialogue, and on empowering participants (first and foremost the researchers and research communities involved) relates to the aims of Callon et al. (2009). In their book *Acting in an Uncertain World,* they claim that technology development is to be regarded as neither rational and inherently historical nor completely dependent of external factors such as price, but rather as guided by socio-cultural, economic and political factors. Governance of science and technology takes too little account that formal and explicit programmes often fail to proactively steer scientific progress and technology innovation. To this aim, a continuous evaluation of objectives, actors and results is necessary. Their need of a less technocratic governance of science and technology follows from their analysis of traditional governance styles as flawed. The aim is non-policy oriented dialogue, which aims primarily to contribute to deliberation and learning among participants, i.e. publics as well as scientists. In other words, governance is considered here as a learning process, less directed to direct intervention and ‘decision-making’, and more towards experimentation. Callon et al. advance the alternative notion of ‘measured action’ or measured decision-making, where “you do not decide [an outcome], you take measures” that are based on inclusive processes that involve both experts and the public, but that ultimately remain open-ended so as to incorporate new knowledge, discoveries, and claims. Such mutual learning is proposed by a plethora of other experts in the field, specifically in Dutch discourse on science policy, including Swierstra’s concept of NEST ethics (Swierstra & Rip 2007), Governance here stops being a means of implementing policy but is instead a process that needs to be collectively done.

Thirdly, on the basis of our study, we see the emergence of new, more hybrid styles of governance, in which the role of expert knowledge is explicitly acknowledged, but the range of relevant forms of expertise is broadened as described by Collins and Evans in the early 2000s. In their famous article ‘The Third Wave of Science Studies’ (2002), they claim that a third wave of science studies is emerging. The first wave concerns the period in which scientific expertise was seen as authoritative and not accessible to non-experts (and therefore esoteric), demanding a ‘top-down approach’ to its policies. The second wave concerns the analysis and sociological deconstruction of the distinction between science and society. This second wave, in their view, went too far in taking a neutral stance in reducing scientific expertise to a social phenomenon like any other social phenomenon, thereby failing to create a perspective for action.

The third wave they see emerging and applaud is a normative turn of this second wave that restores the notion of expertise. This however has not received a follow-up in the RRI approach. Civil society organisations (CSOs) and research bodies need to work together with the view to developing socially desirable products. In this sense, ‘doing governance’ needs a shift from risk governance to innovation governance (Von Schomberg 2011a. This is only possible on the basis of co-responsibility of actors for the whole process and its outcomes, so research priorities can be defined, and knowledge gaps and risks can be identified at the right moment. This, however, requires an entire dissolution of the social-science distinction. This issue has been on the agenda for many years already. Nowotny et al. (2001) were critical of the recurring tendency to delimit the sphere of science from the sphere of society. Also, they were not satisfied with the mere concept of ‘co-evolution’ and attempted to give a more differentiated account of their relation. To do so, Nowotny et al. sketched a distinction between ‘Mode-1’ (disciplinary, predictive and linear) and ‘Mode-2’ (context-driven, problem-focused and interdisciplinary) science. This way, they gave a view of social accountability of knowledge production as a key indicator of scientific quality and scientific reliability.

Whilst addressing the need of policy responsibility over research and innovation, the RRI approach runs the risk of downplaying the responsibility of scientific experts. Thus, the ability of scientists to take responsibility on the basis of their expert authority within the general management of science and technology is decreased. Therefore, we would argue, the acknowledgement of expertise in all its diversity needs to be reinforced in current policy practices.

## Conclusion

In this paper we have considered the emergence and continued occurrence of three styles of governance in science and technology in Europe over the past 30 years. Each of the styles emerged as a response to the increasing democratisation of science governance. First, citizens desired science and technology to be more overtly regulated rather than being largely separate from and unquestioned by society. Secondly, it was acknowledged that ethics review needed to be (seen as) as important as purely scientific and technology risk review. Thirdly, the review of science and technology was not a matter for experts alone; such review was to be seen as a matter where public opinion was equally valid as other voices, and that citizens could speak for themselves in the debate about the meaning and framing of science and technology. However, we also discussed some important shortcomings in these styles of governance.

The styles of governance discussed earlier in this paper share a tendency to take a top-down technocratic role when put into practice, and thus they have a shared pitfall: they either frustrate giving voice to societal views and opinions or become a scapegoat for pre-existing agendas. In line with the authors discussed above, we believe that in defining alternative approaches to the governance of science and technology, any new approach needs to consider governance as an open-ended process. There is neither a silver bullet for governance of science and technology, nor is it possible to design a one-size-fits-all tool to accommodate all normative issues related to science and technology. Open-endedness is key to managing such issues in governance.

RRI seemingly aims for strengthening such open-endedness, but in its departure from traditional styles of governance, it actually closes off many aspects of the process: scientific expertise is made to bow to private interest; advice from independent ethics experts is considered redundant, and the needs and desires of the public are reduced to mere consumer preferences. Strong debate over the true content and meaning of RRI is therefore urgently needed. Different ways of facilitating social criticism may have formal purpose of voicing social and ethical views, to have an influence on policies involved. But since the policy institutes involved are also under pressure of private interests, institutionalising social criticism may be merely a means to silence it.

It is acknowledged that governance of science and technology needs to get away from the rather limited rhetoric of safety. Currently, governance of science and technology is dominated by a risk-safety-and-precaution discourse. This already frames the ethical debate to a restricted series of topics, excluding important moral issues such as justice, welfare standards for marginalised groups, politics of exclusion, privacy, etc. We should not merely ask ‘is it (un)safe', thereby putting this up as the only possible barrier for innovation, prioritising the economic game. We should actually ask, beyond that, ‘is it (un) just?’ Economic progress is not a self-evident guarantee for justice; technologies can be profitable in spite of inefficiency and in spite of possibly associated societal harms. This necessitates a governance discourse that does not restrict itself to the definition and implementation of regulation in the form of negative constraints for science and technology but also of positive aims in a societal setting. This necessitates arranging governance of science and technology in such a way that it serves as an incentive for right-impact-innovations (in the societal sense). This necessitates arranging good governance so that it goes beyond the mere illusion of caring by using participatory devices and ethical expert input as a scapegoat for moral acceptability. Current frameworks for governance direct innovation towards products that yield economic gain, which means products for the more prosperous, dominant groups in European societies, and on a global scale products for the west. Developing countries and marginalised groups do not fall within the scope of governance as a result. Laying this bare, making this transparent as the actual agenda of governance is necessary to address such motivations and counter such effects. Awareness of such underlying agendas is a key element for doing good governance. For a just approach to governance of science and technology, we need to define what we owe to each other, and on what basis.

The arguments for an assessment of the ‘right impacts’ of science and technology in European society find their resonance in the concept of ‘Responsible Research and Innovation’. However, the road towards a mutually responsive interaction should not be treated as a shortcut by which one circumvents moral issues connected to science and technology, but as a means to better account for them. Furthermore, although interaction has been the buzzword for policy for the past 20 years, it also holds its limits: when one is no longer allowed to spend time and energy on reflection, there is no longer anything to interact about.
